# *In vitro* effects of hydrogen peroxide on rat uterine contraction before and during pregnancy

**DOI:** 10.3325/cmj.2018.59.327

**Published:** 2018-12

**Authors:** Rahmah Alanazi, Mohammed Alotaibi, Laiche Djouhri

**Affiliations:** 1 Department of Physiology, College of Medicine, King Saud University, Riyadh, Saudi Arabia; 2 Department of Basic Medical Sciences, College of Medicine, University of Qatar, Doha, Qatar; * RA and MA contributed equally.

## Abstract

**Aim:**

To assess the *in vitro* effect of hydrogen peroxide (H_2_O_2_) on uterine contractions in pregnant and non-pregnant rats.

**Methods:**

The study was performed at the Department of Physiology, College of Medicine, King Saud University from December 2016 to October 2017. Intact uterine samples were obtained from non-pregnant (n = 7-8) and term-pregnant (n = 6-7) rats. Small longitudinal uterine strips were dissected and mounted in an organ bath. Isometric force measurements were used to assess the effect of 400, 800, and 1000 μM H_2_O_2_ on spontaneous uterine contractions and contractions induced by oxytocin (5 nM), high calcium (Ca^+2^) solution (6 mmol/L), and high potassium chloride (KCl) solution (60 mmol/L).

**Results:**

In both term-pregnant and non-pregnant uterine strips, H_2_O_2_ elicited a biphasic response, consisting of a transient contraction followed by a persistent decrease in spontaneously generated contractions, contractions induced by oxytocin, and contractions induced by high Ca^+2^ (all *P* < 0.01, compared with controls) in a concentration-dependent manner. The effect of H_2_O_2_ was more pronounced in non-pregnant than in pregnant rats (*P* < 0.05). In both groups, H_2_O_2_ failed to relax uterine strips pre-contracted with high-KCl solution (*P* > 0.05 compared with controls).

**Conclusion:**

H_2_O_2_ was shown to be a potent uterine relaxant in pregnant and non-pregnant states. The pregnant uterus better withstood the inhibitory effect of H_2_O_2_ than non-pregnant uterus.

Uterine smooth muscles in pregnancy undergo extensive metabolic changes to support the physiological process of labor. At the onset of labor, the relatively quiescent myometria change suddenly to a very excitable tissue producing strong intermittent contractions. These contractions briefly compress the uterine blood vessels, resulting in repetitive ischemia and hypoxia (1,2), which generate reactive oxygen species (ROS), such as superoxide (O_2_^-^), hydrogen peroxide (H_2_O_2_), and peroxynitrite (NO_3_^-^) (3-5). At the same time, uterine smooth muscles produce antioxidant enzymes that minimize the destructive effect of ROS (6). H_2_O_2_ is an important signaling molecule with long half-life in biological systems and the ability to diffuse easily across the plasma membranes (7).

Hypoxia and ischemia have deleterious effects on pH and uterine metabolites, including adenosine 5′-triphosphate and phosphocreatine (1). Our previous study showed that hypoxia significantly decreased or inhibited the force of uterine contraction in rats from different gestation stages (8). At the molecular and cellular level, a uterine contraction is initiated by calcium (Ca^2+^) influx from the extracellular milieu via the voltage-gated calcium channels (VGCCs) or Ca^2+^ release from the sarcoplasmic reticulum (SR). The uterine contraction force during labor can be augmented by oxytocin, which further increases Ca^2+^ influx and release (9).

The contraction force induced by oxytocin was decreased in non-laboring pregnant women by O_2_^-^ and H_2_O_2_ (10). However, different types of smooth muscles have different contractile response to H_2_O_2_. Aortic and airway smooth muscles contract (11,12), whereas smooth muscles of the mesenteric arteries and intestine relax (13,14). Because the contractile responses to H_2_O_2_ differ depending on the species, tissue type, experimental design, and contractile state (quiescent or pre-contracted), no consensus has been reached on the exact effect of H_2_O_2_ on a specific type of smooth muscle. Given that ROS generation within the uterine compartments is a part of the normal muscle contraction and labor process, we hypothesize that excessive ROS production could decrease the force of uterine contractions, which may be pronounced in non-pregnant uterus. The aim of this study was to determine the effects of H_2_O_2_ on spontaneously generated uterine contraction and contractions induced by oxytocin, high extracellular calcium (high-Ca^2+^) solution, and high potassium chloride (KCl) solution, and to examine if the response to H_2_O_2_ is gestationally different.

## Material and methods

### Experimental animals

The experiments included virgin non-pregnant (200 g, n = 7-8) and term-pregnant female Wistar rats (22 days of gestation, n = 6-7). The sample size was determined based on our experience and previous studies (8), which suggested that clear and consistent drug effects on uterine contraction are observed in sample sizes of 6-7. It was also based on the recommendations for the use of minimum number of animals by the UK Animals (Scientific Procedures) Act 1986. The experimental protocol was approved by and carried out according to the Institutional Animal Care Committee (IACC) of King Saud University recommendations (September 2016). The study was performed at the Department of Physiology, College of Medicine, King Saud University from December 2016 to October 2017. The animals were sacrificed by cervical dislocation under CO_2_ anesthesia in accordance with the UK Home Office guidelines (*https://www.legislation.gov.uk/ukpga/1986/14/schedule/1*). The uterus was removed and immediately placed into physiological Krebs saline solution. A longitudinal uterine strip (2 mm ×10 mm) was dissected from each uterus, followed by mechanical removal of the endometrial layer.

### Solutions and chemicals

Krebs solution was composed of the following (in mmol/L): 115 NaCl, 4.7 KCl, 2 CaCl_2_, 1.16 MgSO_4_, 1.18 KH_2_PO_4_, 22 NaHCO_3_, and 7.88 dextrose, pH 7.4. High-KCl solution (60 mmol/L) was prepared by isosmotic substitution of KCl for NaCl. Oxytocin was used at a final concentration of 5 nM and added directly to Krebs solution. High-Ca^2+^ solution was prepared by increasing the extracellular CaCl_2_ concentration in Krebs solution from 2 to 6 mmol/L. H_2_O_2_ was added directly to the Krebs solution. All chemicals and drugs were of analytical grade and purchased from Sigma (St. Louis, MO, USA).

### Isolated tissue bath protocols

The uterine strips for isometric force recordings were prepared as described in our previous study (8). Briefly, isolated uterine strips were tied up from both ends using surgical silk and mounted vertically in a tissue organ bath (Panlab, ADInstruments Ltd, Sydney, Australia). The bath was continuously perfused with a warmed Krebs solution at a rate of 4 mL/min and bubbled with 95% O_2_ and 5% CO_2_ at 37°C. The uterine strips were attached to an isometric force transducer (ADInstruments Ltd) under 1 g resting tension, and the force of contraction was measured in millinewtons. Cumulative concentrations of H_2_O_2_ (400, 800, and 1000 μM) were applied to the intact uterine strips as follows: 1) during spontaneous contraction; 2) during stimulation by oxytocin; 3) during stimulation by high-Ca^2+^ solution; and 4) during stimulation by high-KCl solution. In all experiments, H_2_O_2_ was applied for 20 minutes, after which the tissue was washed out to allow recovery. Each H_2_O_2_ dose was tested on new uterine strips as some uterine strips died or did not recover from the toxic effect of the drug.

### Statistical analysis

Data are expressed as means ± standard deviation (SD), with “n” representing the number of uterine strips, one from each rat. The normality of data distribution was tested using Shapiro-Wilk test and by visual inspection of the histogram and normal Q-Q plots for each H_2_O_2_ concentration. Regular contractile activity in the last 10 minutes in the control Krebs solution (before adding any H_2_O_2_ concentration) was calculated as 100% control. The contractile activity in the last 10 minutes during H_2_O_2_ application was measured and expressed as a percentage of the preceding control period. Force amplitude, frequency (number of contractions in 10 min), and force integral (entire area under the curve, AUC) were compared between two groups using *t* test and between three groups using one-way ANOVA with Bonferroni correction. The level of significance was set at *P* < 0.05. The analysis was performed using OriginLab software (OriginLab, Northampton, MA, USA).

## Results

**Figure 1:**
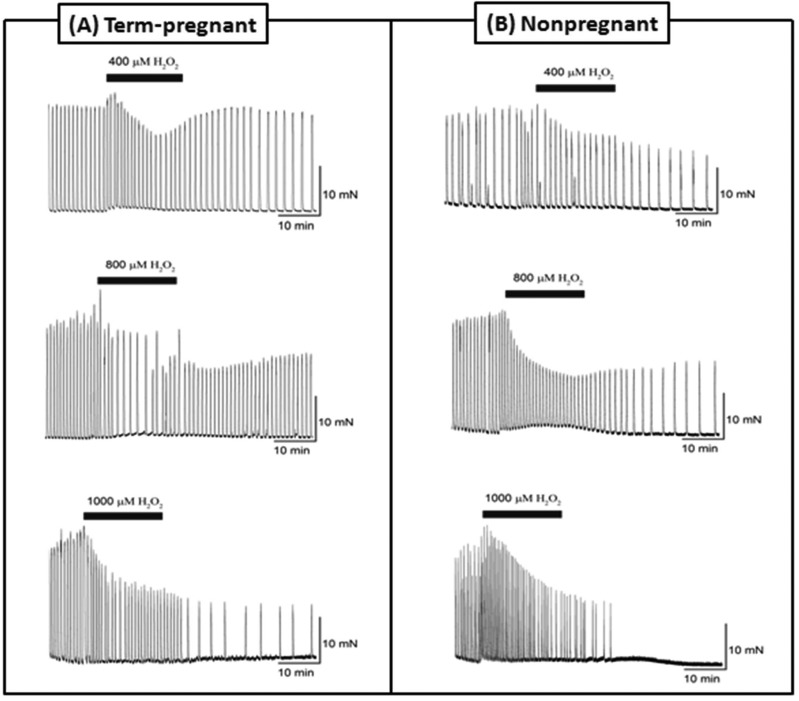
Original recordings showing the contractile responses of uterine strips to 400 μM, 800 μM, and 1000 μM of hydrogen peroxide (H_2_O_2_) during spontaneous activity in (**A**) term-pregnant and (**B**) non-pregnant rats. mN – millinewton.

**Figure 2:**
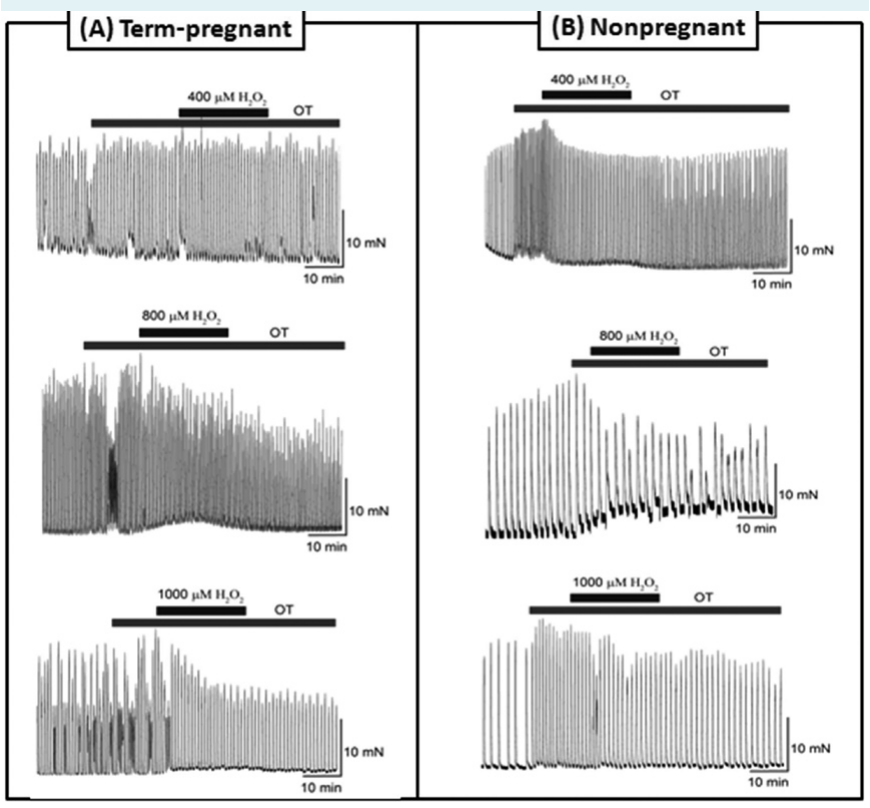
Original recordings showing the contractile responses of uterine strips in the presence of 5 nM oxytocin (OT) to 400 μM, 800 μM, and 1000 μM of hydrogen peroxide (H_2_O_2_) in (**A**) term-pregnant and (**B**) non-pregnant rats. mN – millinewton.

**Figure 3:**
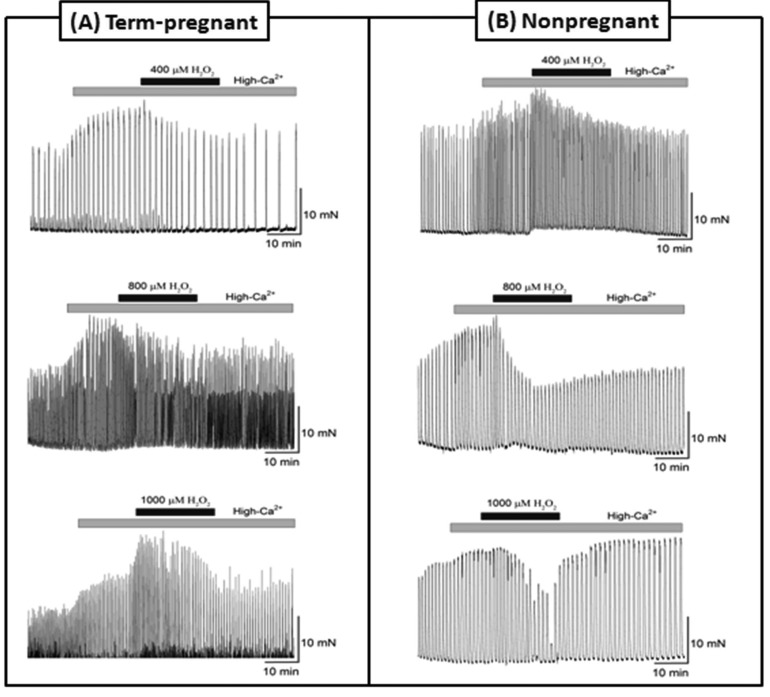
Original recordings showing the contractile responses of uterine strips in the presence of 6 mmol/L extracellular high-calcium (Ca^2+^) to 400 μM, 800 μM, and 1000 μM of hydrogen peroxide (H_2_O_2_) in (**A**) term-pregnant and (**B**) non-pregnant rats. mN – millinewton.

**Figure 4:**
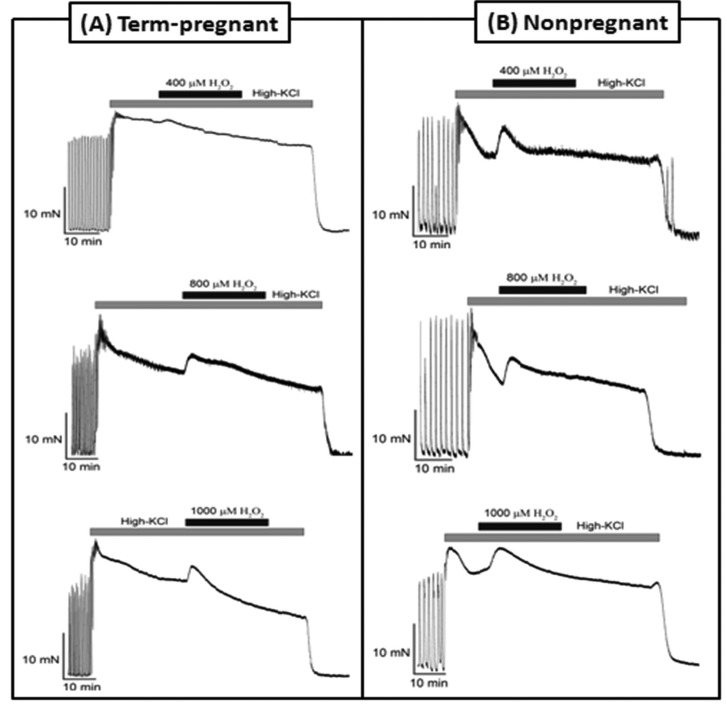
Original recordings showing the contractile responses of uterine strips in the presence of 60 mmol/L potassium chloride (KCl) to 400 μM, 800 μM, and 1000 μM of hydrogen peroxide (H_2_O_2_) in (**A**) term-pregnant and (**B**) non-pregnant rats. mN – millinewton.

Application of 400 μM, 800 μM, and 1000 μM of H_2_O_2_ caused a transient uterine contraction followed by a marked persistent relaxation in both term-pregnant and non-pregnant rat uteri (Figure 1). Pregnant tissues tolerated the effect significantly better than non-pregnant tissues (Table 1). The same effect of all H_2_O_2_ concentrations was observed on oxytocin-induced (Figure 2, Table 2) and high calcium-induced uterine contractions (Figure 3, Table 3). In the case of high KCl-induced contractions, application of 400 μM, 800 μM, and 1000 μM of H_2_O_2_ also caused a transient contraction, but the force decrease was not significant compared with 100% control (Figure 4, Table 4).

**Table 1:** Effects of different concentrations of hydrogen peroxide (H_2_O_2_) *in vitro* on spontaneous contractions of term-pregnant and non-pregnant rat uteri

	H_2_O_2_ concentrations						
	**before adding H_2_O_2_**	**400 μM**	**800 μM**	**1000 μM**	**400 μM**	**800 μM**	**1000 μM**
**Contraction parameters (mean ± standard deviation, %)**	**control**	**term-pregnant (n = 7)**			**non-pregnant (n = 8)**		
**Amplitude**	100	90 ± 3*	70 ± 6*	56 ± 5*	82 ± 3*^†^	63 ± 3*^†^	51 ± 2*^†^
**Frequency**	100	87 ± 8*	75 ± 3*	56 ± 6*	82 ± 8*	63 ± 8*^†^	53 ± 3*
**Area under the curve**	100	84 ± 5*	73 ± 3*	63 ± 3*	75 ± 3*^†^	65 ± 3*^†^	57 ± 3*^†^

*P < 0.01 compared with control (ANOVA/Bonferroni).

†*P* < 0.05 compared with term-pregnant (*t*-test).

**Table 2:** Effects of different concentrations of hydrogen peroxide (H_2_O_2_) *in vitro* on oxytocin-induced contractions in term-pregnant and non-pregnant rat uteri

	H_2_O_2_ concentrations						
	before adding H_2_O_2_	400 μM	800 μM	1000 μM	400 μM	800 μM	1000 μM
Contraction parameter (mean ± standard deviation, %)	control	term-pregnant (n = 6)			non-pregnant (n = 7)		
**Amplitude**	100	82 ± 6*	75 ± 3*	60 ± 3*	78 ± 3*	67 ± 3*^†^	52 ± 3*^‡^
**Frequency**	100	85 ± 6*	69 ± 3*	64 ± 3*	82 ± 6*	67 ± 3*	56 ± 3*^‡^
**Area under the curve**	100	73 ± 6*	64 ± 3*	57 ± 3*	72 ± 3*	64 ± 3*	53 ± 3*^†^

**Table 3:** Effects of different concentrations of hydrogen peroxide (H_2_O_2_) *in vitro* on uterine contractions induced by high-Ca^2+^ solution in term-pregnant and non-pregnant rat uteri

	H_2_O_2_ concentrations						
	before adding H_2_O_2_	400 μM	800 μM	1000 μM	400 μM	800 μM	1000 μM
**Contraction parameters (mean ± standard deviation, %)**	**control**	**term-pregnant (n = 6)**			**non-pregnant (n = 7)**		
**Amplitude**	100	83 ± 3*	67 ± 5*	60 ± 3*	82 ± 3*	61 ± 3*^†^	53 ± 3*^†^
**Frequency**	100	82 ± 6*	67 ± 3*	62 ± 3*	80 ± 3*	62 ± 3*^†^	55 ± 3*^†^
**Area under the curve**	100	83 ± 3*	65 ± 3*	64 ± 3*	83 ± 3*	60 ± 3*^†^	58 ± 3*^†^

*P < 0.01 compared with control (ANOVA/Bonferroni).

†*P* < 0.05 compared with term-pregnant (*t*-test).

**Table 4:** Effects of different concentrations of hydrogen peroxide (H_2_O_2_) *in vitro* on uterine contractions induced by high potassium chloride solution in term-pregnant and non-pregnant rat uteri*

	H_2_O_2_ concentrations						
	before adding H_2_O_2_	400 μM	800 μM	1000 μM	400 μM	800 μM	1000 μM
**Contraction parameters (mean ± standard deviation, %)**	**control**	**term-pregnant (n = 6)**			**non-pregnant (n = 7)**		
**Area under the curve**	100	97 ± 3	98 ± 3	96 ± 6	96 ± 6	98 ± 3	96 ± 8

*There were no significant differences among the three doses of H_2_O_2_ concentrations between the two groups.

## Discussion

H_2_O_2_ decreased uterine contractions induced by different mechanisms in a concentration-dependent manner in both pregnant and non-pregnant rats. However, in comparison with non-pregnant tissue, pregnant tissue tolerated the relaxant effect of H_2_O_2_ better.

H_2_O_2_ has been extensively used to induce experimental oxidative stress in isolated vascular and non-vascular smooth muscles. Our results are in agreement with previous findings on the ability of H_2_O_2_ to significantly decrease oxytocin-induced uterine contraction in pregnant women (10) and uterine contractions generated spontaneously or induced by 6 mmol/L Ca^2+^ in non-pregnant rats (15). H_2_O_2_ exerts its effects through cell membrane ion channels (16), potassium channels (16-19), calcium channels (20), and Ca^2+^-activated Cl^–^ or Na^+^ currents (17).

The observed biphasic response to H_2_O_2_ consisting of an initial transient contraction followed by a persistent relaxation may be explained by Ca^2+^ influx or release by H_2_O_2_. These findings are supported by previous studies in other types of smooth muscles, where H_2_O_2_ application increased intracellular calcium (Ca^2+^)_i_ via either calcium influx from the extracellular space (20) or calcium release from the SR (18). In other studies, blocking Ca^2+^ entry through VGCCs partially blocked H_2_O_2_-induced muscle contraction (11,19). In addition, blocking other Ca^2+^-permeable action channels, such as receptor- and store-operated channels, with a non-selective Ca^2+^ inhibitor markedly decreased [Ca^2+^]_i_ and the contractile response to H_2_O_2_ (11).

Another proposed mechanism of H_2_O_2_-induced transient contraction is the stimulation of prostanoids biosynthesis. Transient contraction induced by H_2_O_2_ is strongly inhibited by blocking prostanoid enzymes, including cyclooxygenases and thromboxane A_2_ (TXA_2_) synthase (21,22), which are expressed by uterine smooth muscles (23,24). Therefore, we cannot exclude the possibility of prostanoids production by H_2_O_2_, which plays an essential role in the uterine activity regulation (25).

The delayed relaxation response to H_2_O_2_ may suggest other molecular mechanisms beyond the membrane channels. H_2_O_2_ could mediate myosin light chain phosphorylation, whose decrease or inhibition causes relaxation response to H_2_O_2_ (26).

High-KCl solution changes the reversal K^+^ potential, depolarizing the membrane, opening the VGCCs, and increasing [Ca^2+^]_i_. In addition, increasing external (K^+^) impairs K^+^ channel function by reducing the driving force for K^+^ efflux, thereby functionally limiting the influence of K^+^ channels on muscle activity (27). In our study, H_2_O_2_ failed to decrease uterine contraction induced by high-KCl, which suggests that H_2_O_2_ may not directly block VGCCs. This is consistent with the results of another study on arterial smooth muscles (28). Therefore, the relaxation response to H_2_O_2_ could be partly mediated by the activation of potassium conductance (hyperpolarization) (27), a mechanism supported by pharmacological studies on arterial smooth muscles (29,30) and electrophysiological studies on other cell types (31,32). Lucchesi et al (33) demonstrated that H_2_O_2_ elicited contraction in smooth muscle of the mesenteric arteries in compromised K^+^ channels (ie, in the presence of high-KCl solution), but that it elicited relaxation in uncompromised K^+^ channels. In the smooth muscle of blood vessels pre-contracted with high-KCl, H_2_O_2_ caused transient contraction dependent on Ca^2+^ influx from the extracellular space (12). We suggest that the relaxation response to H_2_O_2_ in the rat uterus may directly or indirectly involve K^+^ channels activation, as supported by previous reports (10,15). Although H_2_O_2_ transiently increases [Ca^2+^]_i_ via Ca^2+^ influx pathway, high-KCl solution compromises K^+^ equilibrium and prevents repolarization. The existence of different types of K^+^ channels in the myometrium is well documented, and their stimulation is reported to cause myometrial relaxation (34). In smooth muscles of canine trachealis increased [Ca^2+^]_i_ by H_2_O_2_ activated the large conductance calcium-activated potassium channels (BK_Ca_) and promoted muscle relaxation (35). There are also several studies reporting that H_2_O_2_induces muscle relaxation by activating the voltage-gated K^+^ channels (15,36).

Normal uterine contractions are linked to ischemia and hypoxia within the myometrium along with the decrease in energy metabolites (37). In labor, however, uterine contractions increase in intensity, duration, and frequency, causing local hypoxic cycles and increasing the energy demand of the uterus to support the labor process. In this study, pregnant uterine tissues tolerated the effects of H_2_O_2_ better than non-pregnant tissues. This supports our pervious results, which showed that hypoxia decreased rat uterine contraction in different gestational stages, but that the term-pregnant uterus was more resistant to the deleterious effect of hypoxia than non-pregnant uterus (8) owing to pregnancy-related changes in myometrial metabolites and ion channels.

The primary limitation of our study is the death of some uterine tissues caused by the toxic effect of the high H_2_O_2_ dose (1000 μM). In addition, due to financial restrictions, we did not test whether antioxidant agents counteracted the deleterious effects of H_2_O_2_. Another limitation is the small sample size as we had to adhere to the strict IACC guidelines and use the minimum number of animals. However, the sample size was not smaller than those used in other similar studies (10,15). *Post-hoc* power analysis showed that comparison of AUC (1000 μM) between pregnant and non-pregnant animals during spontaneous contraction had an adequate power (0.95 at 5% significance level, G*Power 3.1.9.3, Heinrich-Heine- Universität Düsseldorf, Düsseldorf, Germany) (38), confirming that the number of animals per group was sufficient.

In conclusion, our results show that exogenous H_2_O_2_ causes transient uterine contraction followed by persistent relaxation in both pregnant and non-pregnant rats. The decrease in contraction force was observed in all uterine strips independent of the type of stimulation (spontaneous, oxytocin, high-Ca^2+^). However, when K^+^ channels were blocked by high-KCl, the relaxation response to H_2_O_2_ was inhibited. Further studies are required to unravel the cellular and molecular mechanisms of H_2_O_2_-induced relaxation before, during, and after pregnancy.

## 

**Table:** 

*P < 0.01 compared with control (ANOVA/Bonferroni).
†*P* < 0.05 compared with term-pregnant (*t*-test).
‡*P* < 0.01 compared with term-pregnant (*t*-test).

## References

[1] Larcombe-McDouall J, Buttell N, Harrison N, Wray S (1999). In vivo pH and metabolite changes during a single contraction in rat uterine smooth muscle. ‎. J Physiol.

[2] Brar HS, Platt LD, DeVore GR, Horenstein J, Medearis AL (1988). Qualitative assessment of maternal uterine and fetal umbilical artery blood flow and resistance in laboring patients by Doppler velocimetry.. Am J Obstet Gynecol.

[3] Woods JR, Cavanaugh JL, Norkus EP, Plessinger MA, Miller RK (2002). The effect of labor on maternal and fetal vitamins C and E.. Am J Obstet Gynecol.

[4] Nakai A, Oya A, Kobe H, Asakura H, Yokota A, Koshino T (2000). Changes in maternal lipid peroxidation levels and antioxidant enzymatic activities before and after delivery.. J Nippon Med Sch.

[5] Zyrianov V, Sumovskaya AY, Shostak A (2003). Application of electron spin resonance for evaluation of the level of free radicals in the myometrium in full-term pregnancy with normal labour and uterine inertia.. J Biosci.

[6] Khan RN, Matharoo-Ball B, Shaw RW (2010). Antioxidant enzyme expression, lipid peroxidation, and protein oxidation in human myometrium with parturition.. Reprod Sci.

[7] Liu X, Zweier JL (2001). A real-time electrochemical technique for measurement of cellular hydrogen peroxide generation and consumption: evaluation in human polymorphonuclear leukocytes.. Free Radic Biol Med.

[8] Alotaibi M, Arrowsmith S, Wray S (2015). Hypoxia-induced force increase (HIFI) is a novel mechanism underlying the strengthening of labor contractions, produced by hypoxic stresses.. Proc Natl Acad Sci U S A.

[9] Gimpl G, Fahrenholz F (2001). The oxytocin receptor system: structure, function, and regulation.. Physiol Rev.

[10] Warren AY, Matharoo-Ball B, Shaw RW, Khan RN (2005). Hydrogen peroxide and superoxide anion modulate pregnant human myometrial contractility.. Reproduction.

[11] Kojima K, Kume H, Ito S, Oguma T, Shiraki A, Kondo M (2007). Direct effects of hydrogen peroxide on airway smooth muscle tone: roles of Ca 2+ influx and Rho-kinase.. Eur J Pharmacol.

[12] Thakali K, Davenport L, Fink GD, Watts SW (2006). Pleiotropic effects of hydrogen peroxide in arteries and veins from normotensive and hypertensive rats.. Hypertension.

[13] Choi S, Yeum CH, Kim YD, Park CG, Kim MY, Park JS (2010). Receptor tyrosine and MAP kinase are involved in effects of H2O2 on interstitial cells of Cajal in murine intestine.. J Cell Mol Med.

[14] Fujimoto S, Asano T, Sakai M, Sakurai K, Takagi D, Yoshimoto N (2001). Mechanisms of hydrogen peroxide-induced relaxation in rabbit mesenteric small artery.. Eur J Pharmacol.

[15] Appiah I, Milovanovic S, Radojicic R, Nikolic-Kokic A, Orescanin-Dusic Z, Slavic M (2009). Hydrogen peroxide affects contractile activity and anti-oxidant enzymes in rat uterus.. Br J Pharmacol.

[16] Wang H, Joseph JA (2000). Mechanisms of hydrogen peroxide-induced calcium dysregulation in PC12 cells1.. Free Radic Biol Med.

[17] Schlief T, Heinemann SH (1995). H2O2-induced chloride currents are indicative of an endogenous Na (+)-Ca2+ exchange mechanism in Xenopus oocytes. ‎. J Physiol.

[18] Lin M-J, Yang X-R, Cao Y-N, Sham JS (2007). Hydrogen peroxide-induced Ca2+ mobilization in pulmonary arterial smooth muscle cells.. Am J Physiol Lung Cell Mol Physiol.

[19] Santiago E, Contreras C, García-Sacristán A, Sánchez A, Rivera L, Climent B (2013). Signaling pathways involved in the H2O2-induced vasoconstriction of rat coronary arteries.. Free Radic Biol Med.

[20] Chaplin NL, Amberg GC (2012). Hydrogen peroxide mediates oxidant-dependent stimulation of arterial smooth muscle L-type calcium channels.. Am J Physiol Cell Physiol.

[21] Gao Y, Lee R (2001). Hydrogen peroxide induces a greater contraction in mesenteric arteries of spontaneously hypertensive rats through thromboxane A2 production.. Br J Pharmacol.

[22] Thakali K, Davenport L, Fink GD, Watts SW (2007). Cyclooxygenase, p38 mitogen-activated protein kinase (MAPK), extracellular signal-regulated kinase MAPK, Rho kinase, and Src mediate hydrogen peroxide-induced contraction of rat thoracic aorta and vena cava.. J Pharmacol Exp Ther.

[23] Moore F, Asboóth G, Loópez Bernal A (2002). Thromboxane receptor signalling in human myometrial cells.. Prostaglandins Other Lipid Mediat.

[24] Swanson ML, Lei ZM, Swanson PH, Rao CV, Narumiya S, Hirata M (1992). The expression of thromboxane A<inf>2</inf>synthase and thromboxane A<inf>2</inf>receptor gene in human uterus.. Biol Reprod.

[25] Erkinheimo T-L, Saukkonen K, Narko K, Jalkanen J, Ylikorkala O, Ristimäki A (2000). Expression of cyclooxygenase-2 and prostanoid receptors by human myometrium.. J Clin Endocrinol Metab.

[26] Fujimoto S, Mori M, Tsushima H (2003). Mechanisms underlying the hydrogen peroxide-induced, endothelium-independent relaxation of the norepinephrine-contraction in guinea-pig aorta.. Eur J Pharmacol.

[27] Barlow RS, White RE (1998). Hydrogen peroxide relaxes porcine coronary arteries by stimulating BKCachannel activity.. Am J Physiol.

[28] Rogers PA, Dick GM, Knudson JD, Focardi M, Bratz IN, Swafford AN (2006). H2O2-induced redox-sensitive coronary vasodilation is mediated by 4-aminopyridine-sensitive K+ channels.. Am J Physiol Heart Circ Physiol.

[29] Ward CA, Giles WR (1997). Ionic mechanism of the effects of hydrogen peroxide in rat ventricular myocytes. ‎. J Physiol.

[30] Hayabuchi Y, Nakaya Y, Matsuoka S, Kuroda Y (1998). Hydrogen peroxide-induced vascular relaxation in porcine coronary arteries is mediated by Ca 2+-activated K+ channels.. Heart Vessels.

[31] Seutin V, Scuvée-Moreau J, Massotte L, Dresse A (1995). Hydrogen peroxide hyperpolarizes rat CA1 pyramidal neurons by inducing an increase in potassium conductance.. Brain Res.

[32] Filipovic DM, Reeves WB (1997). Hydrogen peroxide activates glibenclamide-sensitive K+ channels in LLC-PK1 cells.. Am J Physiol.

[33] Lucchesi PA, Belmadani S, Matrougui K (2005). Hydrogen peroxide acts as both vasodilator and vasoconstrictor in the control of perfused mouse mesenteric resistance arteries.. J Hypertens.

[34] Khan RN, Matharoo-Ball B, Arulkumaran S, Ashford ML (2001). Potassium channels in the human myometrium.. Exp Physiol.

[35] Janssen LJ, Netherton SJ, Walters DK (2000). Ca 2+-dependent K+ channels and Na+-K+-ATPase mediate H 2 O 2-and superoxide-induced relaxations in canine trachealis.. J Appl Physiol.

[36] Park SW, Noh HJ, Sung DJ, Kim JG, Kim JM, Ryu S-Y (2015). Hydrogen peroxide induces vasorelaxation by enhancing 4-aminopyridine-sensitive Kv currents through S-glutathionylation.. Pflugers Arch.

[37] Harrison N, Wray S, Larcombe-Mcdouall JBA (1995). 31P NMR investigation into the effects of repeated vascular occlusion on uterine metabolites, intracellular pH and force, in vivo.. NMR Biomed.

[38] Faul F, Erdfelder E, Lang A-G, Buchner AG (2007). * Power 3: A flexible statistical power analysis program for the social, behavioral, and biomedical sciences.. Behav Res Methods.

